# A super-SILAC based proteomics analysis of diffuse large B-cell lymphoma-NOS patient samples to identify new proteins that discriminate GCB and non-GCB lymphomas

**DOI:** 10.1371/journal.pone.0223260

**Published:** 2019-10-11

**Authors:** L. E. van der Meeren, J. Kluiver, B. Rutgers, Y. Alsagoor, P. M. Kluin, A. van den Berg, L. Visser

**Affiliations:** 1 Department of Pathology and Medical Biology, University of Groningen, University Medical Centre Groningen, Groningen, The Netherlands; 2 Department of Pathology, University Medical Centre Utrecht, Utrecht, The Netherlands; University of Nebraska Medical Center, UNITED STATES

## Abstract

Diffuse large B-cell lymphoma—not otherwise specified (DLBCL-NOS) is a large and heterogeneous subgroup of non-Hodgkin lymphoma. DLBCL can be subdivided into germinal centre B-cell like (GCB) and activated B-cell like (ABC or non-GCB) using a gene-expression based or an immunohistochemical approach. In this study we aimed to identify additional proteins that are differentially expressed between GCB and non-GCB DLBCL. A reference super-SILAC mix, including proteins of eight B-cell lymphoma cell lines, was mixed with proteins isolated from seven non-GCB DLBCL and five GCB DLBCL patient tissue samples to quantify protein levels. Protein identification and quantification was performed by LC-MS. We identified a total of 4289 proteins, with a four-fold significant difference in expression between non-GCB and GCB DLBCL for 37 proteins. Four proteins were selected for validation in the same cases and replication in an independent cohort of 47 DLBCL patients by immunohistochemistry. In the validation cohort, we observed a non-significant trend towards the same differential expression pattern as observed in the proteomics. The replication study showed significant and consistent differences for two of the proteins: expression of glomulin (GLMN) was higher in GCB DLBCL, while expression of ribosomal protein L23 (RPL23) was higher in non-GCB DLBCL. These proteins are functionally linked to important pathways involving MYC, p53 and angiogenesis. In summary, we showed increased expression of RPL23 and decreased expression of GLMN in non-GCB compared to GCB DLBCL on purified primary DLBCL patient samples and replicated these results in an independent patient cohort.

## Introduction

Gene-expression profiling (GEP) has aided our understanding of the pathogenesis of diffuse large B-cell lymphoma not otherwise specified (DLBCL-NOS) by discriminating two distinct entities; germinal centre B-cell like (GCB) DLBCL and activated B-cell like (ABC) or non-GCB DLBCL. [1–3] Non-GCB DLBCL arises from post-germinal B-cells that are blocked during plasmocytic differentiation. [3] Several immunohistochemistry-based algorithms have been developed to classify DLBCL into non-GCB and GCB subgroups. [4–7] The Hans algorithm is the most commonly used approach to classify DLBCL cases in the routine diagnostic setting. [8] So far, most proteomics studies focused on DLBCL cell lines with some exceptions using primary DLBCL tissue samples. [9–11] Deeb et al. were the first to characterise DLBCL cell lines with super-SILAC, a quantitative proteomics approach, to differentiate between non-GCB and GCB DLBCL. [9] They defined a list of 55 proteins to segregate non-GCB and GCB DLBCL cell lines. We applied super-SILAC on purified tumour cells in search for novel proteins that can further discriminate between non-GCB and GCB DLBCL subgroups. [12] Proteomics results were validated in the same cohort and replicated in an independent cohort of DLBCL patient samples by immunohistochemistry. Thus we focused only on the proteins for which reliable immunohistochemical assays were available.

## Materials and methods

### Selection of cases

We collected viably frozen cell suspensions of 59 DLBCL cases in the tissue bank of the department of Pathology UMCG between 1999 and 2012. All cases were reviewed based on the 2008 WHO classification (Table 1). [13] Based on an initial estimation using H&E staining and immunohistochemistry on paraffin and frozen tissue sections, 31/59 cases were selected based on sufficient numbers of tumour cells (approximately ≥80% tumour) for proteomics analysis. From these cases, sufficient viable single cell suspensions were available for 13 cases. In one case, purification procedures (see below) did not result into a sufficient enrichment of tumour cells, and this case was omitted from further studies. An independent cohort of 47 DLBCL-NOS cases (Table 2) was retrieved from the tissue bank of the department of Pathology UMCG between 1999 and 2012 based on the same criteria mentioned above for replication by immunohistochemistry. This patient cohort has been used in an earlier study. [14] The study protocol was consistent with international ethical and professional guidelines (the Declaration of Helsinki and the International Conference on Harmonization Guidelines for Good Clinical Practice). Approval for this study was obtained from the local ethics review board of the Pathology department of the University Medical Centre Groningen. The Medical ethics review board waives the need for approval if rest material is used under law in the Netherlands and waives the need for informed consent when patient anonymity is assured.

**Table 1:** Features of DLBCL-NOS patients used for super-SILAC.

Patient	Hans	Visco	Age	Sex	Ann Arbor stage	IPI
1	non-GCB	non-GCB	51	Male	3	1
2	non-GCB	non-GCB	78	Male	3	4
3	non-GCB	ND	77	Male	1	2
4	GCB	GCB	52	male	4	2
5	non-GCB	non-GCB	61	Female	3	2
6	non-GCB	non-GCB	66	Male	4	3
7	non-GCB	non-GCB	71	Male	1	1
8	GCB	GCB	66	Male	3	2
9	GCB	GCB	14	Male	1	unknown
10	GCB	ND	48	Female	4	3
11	GCB	GCB	53	Female	4	2
12	GCB	non-GCB	58	Male	1	unknown
13	non-GCB	ND	78	Male	4	3

**Table 2:** Clinical features of the DLBCL-NOS replication cohort.

Characteristics	non-GCB (20)*	GCB (27)*
male (%)	11 (55)	15 (56)
median age (range)	66 (9–85)	56 (20–76)
**Ann Arbor Stage**		
I/II (%)	4 (20)	12 (44)
III/IV (%)	14 (70)	9 (33)
unknown (%)	2 (10)	6 (23)
**IPI**		
0–1 (%)	2 (10)	11 (41)
2–3 (%)	10 (50)	7 (26)
4–5 (%)	5 (25)	1 (4)
unknown (%)	3 (15)	8 (29)

*According to Hans classification

### Immunohistochemistry

Formalin fixed, paraffin embedded (FFPE) tissues were stained for CD10 (Rabbit, clone SP67), BCL6 (mouse, clone CI191E/A8), MUM1 (mouse, clone MUM-1p) by Ventana (Roche, Tucson, USA) and were considered positive when >30% of the tumour cells stained positive. FoxP1 (Abcam, Cambridge, UK) was considered positive when >80% of the tumour cells stained positive. P53 (Abcam, Cambridge, UK) was scored as mutated when the staining was homogeneous positive or negative, and wildtype when variable staining was present. MYC (Ventana, Roche, Tucson, USA) was scored in percentages and no cut-off was used. The cases were classified based on the Hans algorithm using CD10, BCL6 and IRF4/MUM1 (cut off level 30% of the tumour cells) into either non-GCB DLBCL or GCB DLBCL (Tables 1 and 2). [4] For validation and replication analyses of the proteomics results we also applied the Visco 3-thiered algorithm with CD10, FoxP1 and BCL6 (cut off levels 30%, 60% and 30%).

For validation and replication of the proteomics results FFPE tissue sections were stained for GLMN (ab170776 (Abcam, Cambridge, UK)), ADK (HPA038391, Sigma-Aldrich, Darmstadt, Germany), ARMC6 (HPA041420, Sigma-Aldrich, Darmstadt, Germany), and RPL23 (HPA003373, Sigma-Aldrich) (S1 Table). For all stainings, reactive tonsils and other external controls were used as indicated by the manufacturer, publications on these antibodies, as well as control tissues selected from the Protein Atlas (https://www.proteinatlas.org/). Additionally, in all individual slides staining patterns of cells in the microenvironment were used as internal negative and positive controls. All immunohistochemical stainings were independently scored by LM and PK and discrepancies were discussed at a multi-head microscope. Tumour cells were scored in four categories using predefined thresholds based on the number of moderate to strong positive cells: 0%, 1–40%, 41–70% and 71–100%. Thus, cases with a very weak staining in all tumour cells were scored as 0%. In all cases a low percentage of the cells should show some staining, completely blank cases were considered as non-determinable. For statistical analysis we made two categories: cases with 0–40% positive tumour cells were considered as “negative” and cases with 41–100% positive tumour cells as “positive”. In rare borderline cases with a heterogeneous staining intensity, a very strong staining intensity of a subpopulation of tumour cells led to a positive categorization.

### Purification of tumour cells

Tumour cells were isolated using Dynabeads^®^ CD19 Pan B (number 11143D, Thermo Fisher Scientific, Waltham MA, USA) combined with DETACHaBEAD^®^ CD19 (number 12506D, Thermo Fisher Scientific, Waltham MA, USA). Subsequently, the cell suspensions were depleted of naïve B-cells using anti-IgD coated Dynabeads (the tumour cells were IgD negative as assessed before by immunohistochemistry on frozen tissue sections). The purity of the tumour cell fraction was checked by flow cytometry for expression of CD20, κ and λ (IQ Products, Groningen, The Netherlands). After purification, cells were washed three times with cold PBS and centrifuged at 1200 rpm for 5 minutes at 4°C, resuspended in lysis buffer (Cell signalling technologies, Danvers, USA, #9803) and placed on ice for 30–45 min. The supernatant containing mostly membrane and cytoplasmic proteins (nuclei are not efficiently lysed in this buffer) was collected by centrifugation at 14.000 rpm for 10 minutes at 4°C and 20-fold concentrated with the Vivaspin^®^ 2 Centrifugal Concentrator. The protein concentration was measured using the Pierce^™^ BCA Protein Assay Kit (#23227; Thermo Scientific, Waltham MA, USA).

### Culturing cell lines for super-SILAC

Eight B-cell lymphoma cell lines (DSMZ, Braunschweig, Germany) were selected for generating the super-SILAC reference sample; SC-1, DoHH2 (transformed follicular lymphoma), OCI-LY3, U2932 (non-GCB DLBCL), SU-DHL-5, SU-DHL-6, SU-DHL-10, and SU-DHL-4 (GCB DLBCL). The cell lines were cultured in RPMI (Thermo Fisher) and penicillin/streptomycin (P/S) with either 10% fetal bovine serum (FBS) (OCILY3, SC-1, DoHH2 and SU-DHL-4) or 20% FBS (SU-DHL-5, SU-DHL-6, SU-DHL-10) supplemented with heavy ^13^C_6_ L-Lysine-2HCl (Thermo Scientific, prod #88431) and ^13^C_6_^15^N_4_ L-Arginine-HCl (Thermo Scientific prod #88434). All cell lines were tested negative for mycoplasma. Cells were cultured for approximately 10 cell passages to allow maximum incorporation of the labelled amino acids in all proteins. [15–16] The incorporation was checked with mass spectrometry for each individual cell line. After confirmation of sufficient incorporation, cell lines were lysed as described above and 20-fold concentrated with the Vivaspin^®^ 2 Centrifugal Concentrator. The final protein concentration was measured using a BCA protein assay.

### Generation of super-SILAC protein mixes

The cell lines were mixed at equal protein amounts and used as an internal standard, to allow relative quantification of the protein amount and direct comparison of the protein levels obtained for each primary DLBCL sample. The unlabelled patient samples were mixed 1:1 with the super-SILAC mix (50μg:50μg protein). Samples were heated for 5 min at 100°C before SDS-PAGE gel electrophoresis (2 hours, 110V). Each protein lane was divided into 15 equal pieces. Each piece was washed twice in 400 μl MilliQ water for 15 min, twice in ± 400 μl 50% acetonitrile (ACN) and once with 400 μl 100% ACN for 15 min. After removing ACN, 100 μl 10 mM dithiothreitol (DTT) (made in 100 mM ammonium bicarbonate pH 8–8.5) was added and incubated for 1 hour at room temperature. DTT was discarded and the gel pieces were covered with 100 μl 55 mM iodoacetamide (made in 100 mM ammonium bicarbonate pH 8–8.5) and incubated for 45 min at room temperature in the dark. The gel pieces were washed once with 400 μl MilliQ for 15 min, twice with 400 μl 50% ACN for 15 min and once with ± 400 μl 100% ACN for 15 min. After washing the gel pieces, 40 μl 5 ng/μl trypsin solution (made in 20 mM ammonium bicarbonate pH 8–8.5) was added and incubated for 15 min to rehydrate the gel pieces. The trypsin solution was removed and 50 μl 20 mM ammonium bicarbonate pH 8–8.5 was added to cover the gel pieces fully and digested overnight at 37°C. To extract the peptides 1 μl 100% formic acid was added and incubated at room temperature for 5 min (on shaker). Finally, the gel pieces were centrifuged for 1 min on 5000 rpm and the peptide containing supernatant was collected.

### Protein identification

All samples were analysed on the Orbitrap LC-MS (Thermo Fisher Scientific, Waltham MA, USA) and data were analysed with the PEAKS proteomics software platform. ProID 1.1 software (Applied Biosystems, Foster City, CA) [17] was used to predict the corresponding proteins according to the Swiss-Prot database. [18] The list with predicted proteins was collapsed to generate a list with unique proteins. Only proteins with a -10igP of at least 50 and coverage by at least 2 peptides were considered. The differences in protein expression levels are indicated by the heavy/light ratio of each protein relative to the protein amount in the super-SILAC mix.

### Data analysis of non-GCB DLBCL and GCB DLBCL

Data analysis was done with log2-transformed ratios, without normalization or baseline transformation, using GeneSpring GX software (version 14.9, Agilent Genomics, Santa Clara CA, USA). Missing protein ratios were left blank. Proteins were filtered with the criterion that at least 6 out of 7 conditions for the non-GCB DLBCL group or 4 out of 5 conditions for the GCB DLBCL group should have expression values above the background.

### Western blot

Twenty million cells were washed with PBS and lysed in RIPA buffer (50 mM Tris/ 150 mM NaCl/ 2.5 mM Na2EDTA/1% Triton X-100, 0.5% mM sodium deoxycholate/0.1% SDS in dH_2_0) with 1 mM phenylmethanesulphonyl fluoride for 30–45 minutes on ice. Protein concentration was determined using the BCA Protein Assay Kit. Samples were loaded at 40 μg per lane and electrophoresis and blotting was performed according to standard protocols. The antibodies used for immunohistochemistry, were also used for western blotting, except ADK which was not suited for western blot, as described in the supplementary material. Staining with primary antibodies for ARMC6, GLMN and RPL23 was done overnight and staining for GAPDH (1:20,000; clone 6C5 cat nr. 600–502, Novus bio, Centennial CO, USA) was done for one 1 hour at 4°C.

### Statistical analysis

A student’s t-test without multiple testing correction was performed to identify differentially expressed proteins in the proteomics data. In addition, we applied a 4-fold difference to select the most promising candidates. The chi-square test for trend was used (i.e. linear-by-linear association test) for validation and replication immunohistochemistry. P-values <0.05 were considered significant.

## Results

### Selection of cases used for proteomics

Viably frozen cells of the 13 cases were successfully purified, and the purity of the tumour cell fraction as determined by CD20 varied between 85 and 99%. These cases were classified as 6 GCB and 7 non-GCB type DLBCL. The purity as assessed for surface kappa/lambda immunoglobulin expression in flow cytometry was more difficult to interpret due to the presence of many apoptotic cells in two of the cases and an unspecific background staining due to the relatively mild washing procedure applied to the fragile tumour cells. One case with 45% polyclonal B cells was omitted from further analysis. In the remaining 12 cases, the percentage of polyclonal B-cells in the samples ranged from 0–23% with a median of 8% (S2 Table).

### Proteomics analysis

The total number of unique proteins identified in the patient samples was 4289. The number of proteins detected per sample ranged from 2273 to 3154. After filtering for proteins detected in at least 4/5 GCB or 6/7 non-GCB DLBCL cases 2059 proteins remained for further analysis. A significant difference between GCB and non-GCB DLBCL was observed for 132 proteins, of which 37 proteins showed an at least 4-fold difference. Of the three proteins used in the Hans algorithm, only MUM1/IRF4 was included in the list of 2059 proteins with a slightly higher ratio in non-GCB DLBCL patient samples. BCL6, which is also almost exclusively localized in the nucleus, was not detected due to the choice of the lysis buffer; peptides for CD10 were not detected.

**Fig 1:**
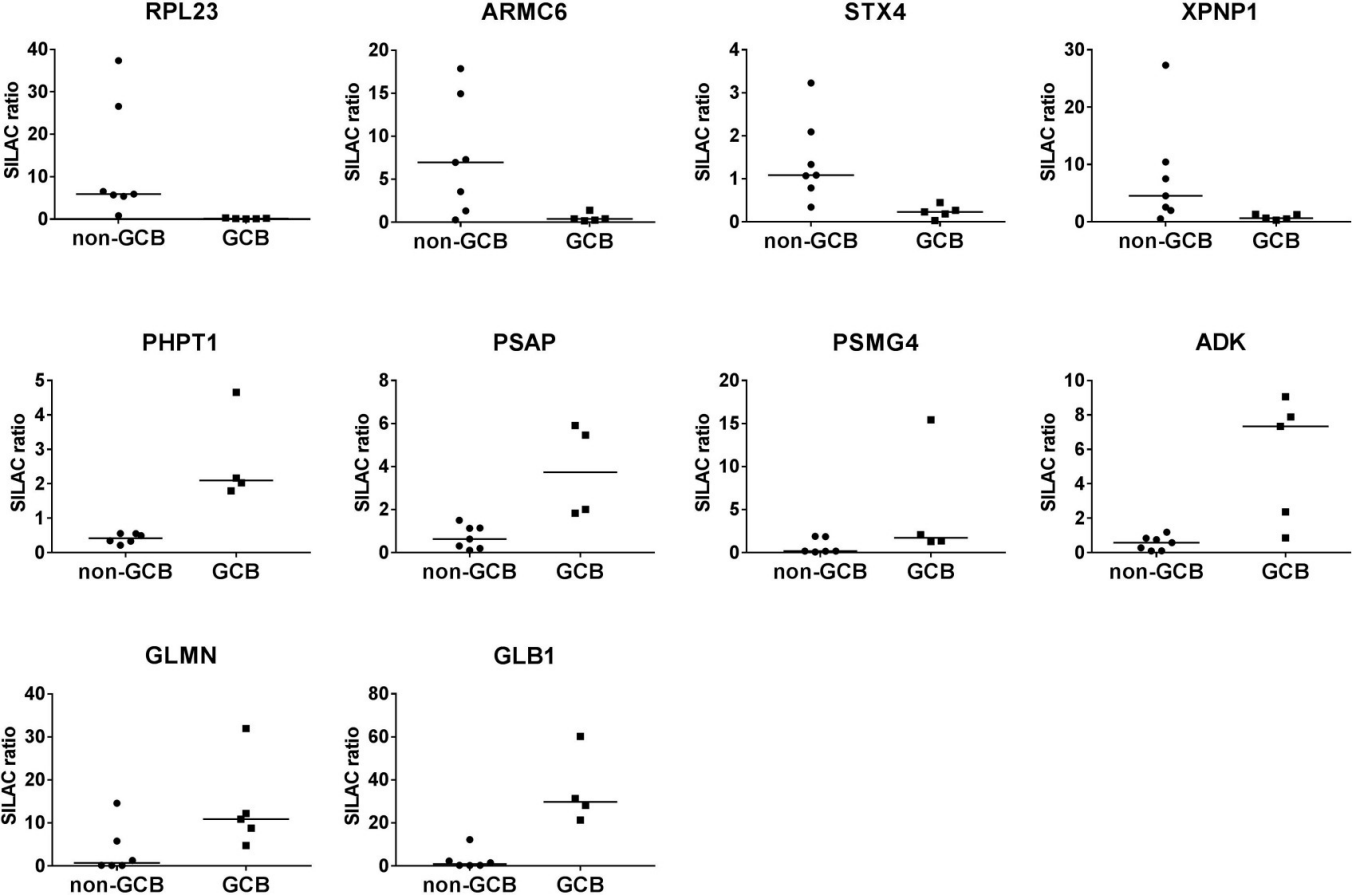
Overview of the super-SILAC ratios for the 4 proteins (from high to low fold change), RPL23 (FC 48,4), ARMC6 (FC 10,4), STX4 (FC 6,9), XPNP1 (FC 6,2)) with higher expression in non-GCB and for the 6 proteins (from low to high fold change)(PHPT1 (FC 6,2), PSAP (FC 6,5), PSMG4 (FC 8,2), ADK (FC 10,5), GLMN (FC 15,2), GLB1 (FC 32,9)), with higher expression in GCB DLBCL with on the Y-axis SILAC ratio (FC, fold change).

Ten of the proteins showed an at least 6-fold difference (Fig 1). Four proteins, i.e. RPL23 (FC 48,4), ARMC6 (FC 10,4), STX4 (FC 6,9) and XPNP1 (FC 6,2), showed higher levels in non-GCB DLBCL cases. The other six proteins, respectively GLB1 (FC 32,9), GLMN (FC 15,2), ADK (FC 10,5), PSMG4 (FC 8,2), PSAP (FC 6,5), PHPT1 (FC 6,2), showed higher levels in GCB DLBCL cases (Fig 1). We selected the 4 proteins with a fold change more than 10 for validation and replication and for which reliable antibodies were available. Ribosomal Protein L23 (RPL23) showed the most pronounced difference with more than 48-fold higher ratios in non-GCB DLBCL (range 0.834 to 37.343), while GCB DLBCL cases showed low to very low expression levels (0.070 to 0.318). Armadillo repeat-containing protein 6 (ARMC6) levels showed 10-fold higher ratios in non-GCB DLBCL (0.273 to 14,948) as compared to GCB DLBCL cases (0.168 to 1.396). Glomulin (GLMN) showed >15 fold higher in GCB DLBCL cases (4.739 to 12.188) compared to non-GCB (0 to 14.596). ADK ratios were 10-fold higher in GCB DLBCL, albeit with marked heterogeneity: low levels in 2 patient samples (0.856 and 2.360) and high ratios in the 3 other patients (9.063, 7.886 and 7.334). The ratios in non-GCB DLBCL patients varied between 0.093 and 1.184 (S3 Table).

### Selection of antibodies and western blot

We selected antibodies that were suitable for immunohistochemistry and raised against protein regions that were covered by peptides in the proteomics analysis. For all four selected proteins, peptides identified in the proteomics analysis mapped along the entire protein. All antibodies were polyclonal and raised against aa505-533 of GLMN, aa10-88 of RPL23, aa44-140 of ARMC6 and aa89-170 of ADK. These regions were always covered by two or more peptides in our patient samples.

**Fig 2:**
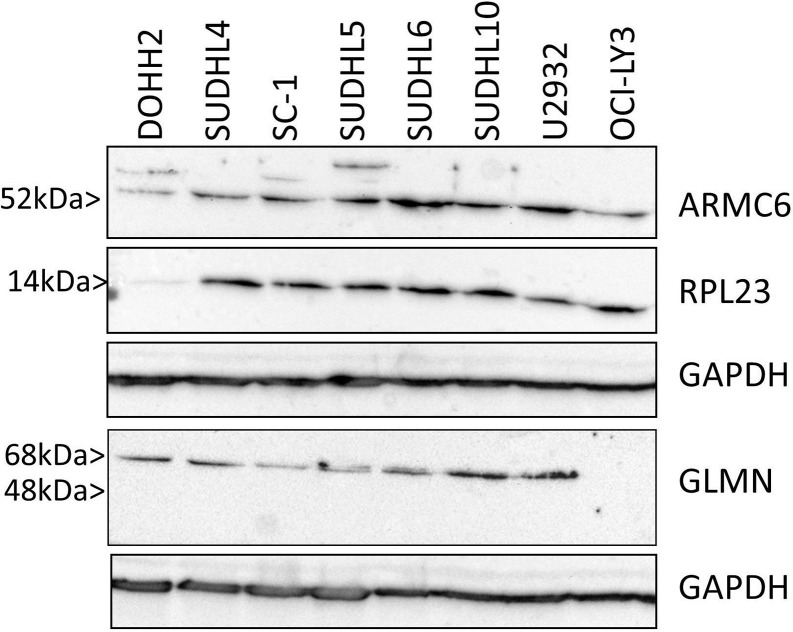
Western blot of three differentially expressed proteins in the cell lines used for to generate the super-SILAC protein mix. ARMC6, RPL23, GLMN, for 8 DLBCL cell lines, from left to right: DoHH2, SU-DHL-4, SC-1, SU-DHL-5, SU-DHL-6, SU-DHL-10, U2932, OCI-LY3.

Western blot analysis was performed to check the correct molecular weight as detected by the selected antibodies and to check expression of the proteins in the cell lines used to generate the super-SILAC protein mix. In addition, we wanted to check which isoform, either 48 kDa or 68kDa of GLMN was expressed in the lymphoma cell lines, as the antibody cannot distinguish between both isoforms, FAP48 and FAP68, which have different functions. GLMN was expressed in all cell lines except for OCI-LY3 and showed a molecular weight of 68kDa. ARMC6 was expressed in all cell lines. RPL23 protein was detected in all cell lines but DoHH2. (Fig 2).

### Validation of the proteomics results

**Fig 3:**
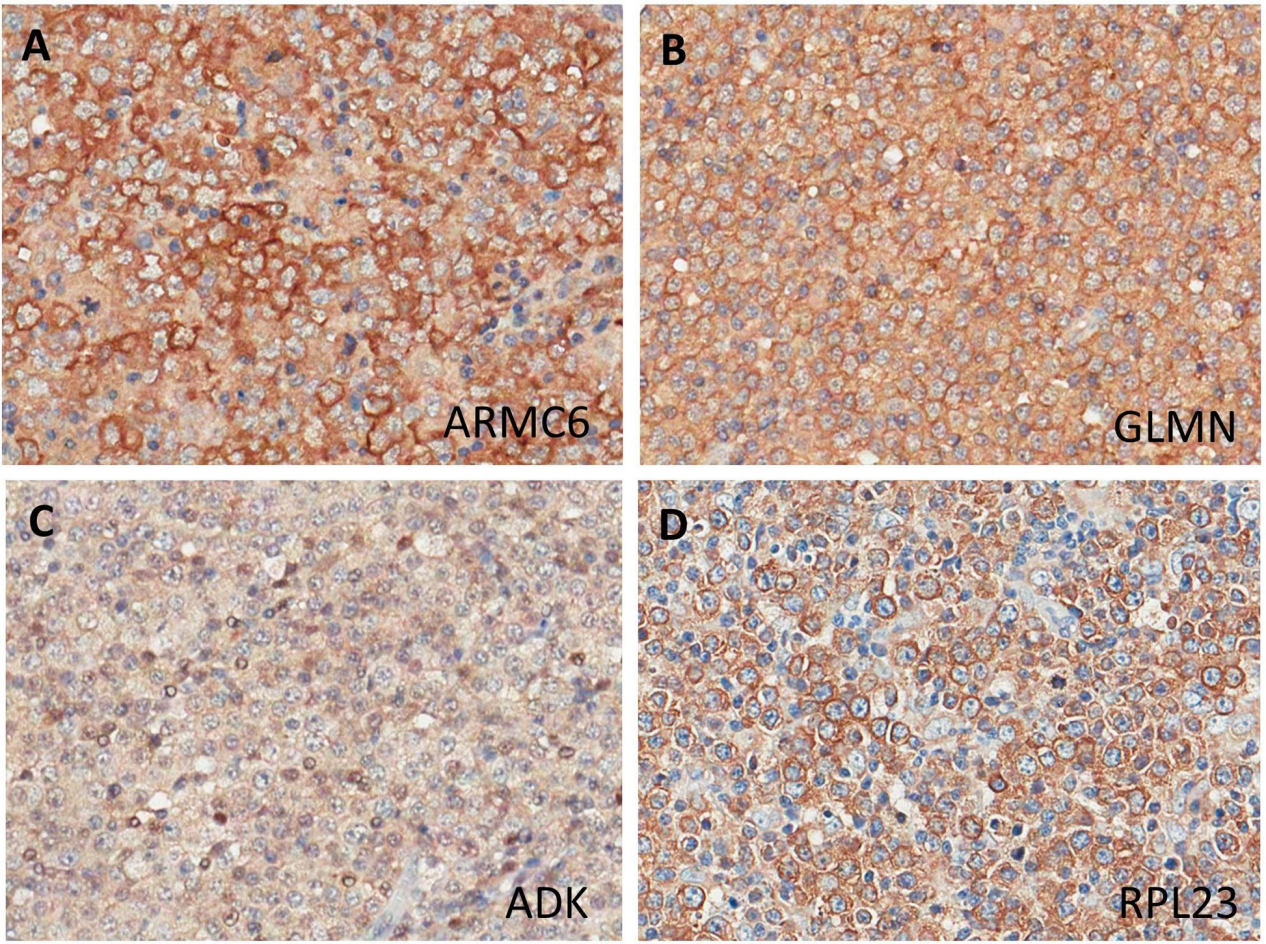
Representative images of the immunohistochemical staining results of the four selected proteins in GCB and non-GCB DLBCL. ARMC6 (non-GCB case), GLMN (GCB case) and RPL23 (non-GCB case) showed cytoplasmic expression and ADK (GCB case) staining in this case was observed only in the nucleus.

**Fig 4:**
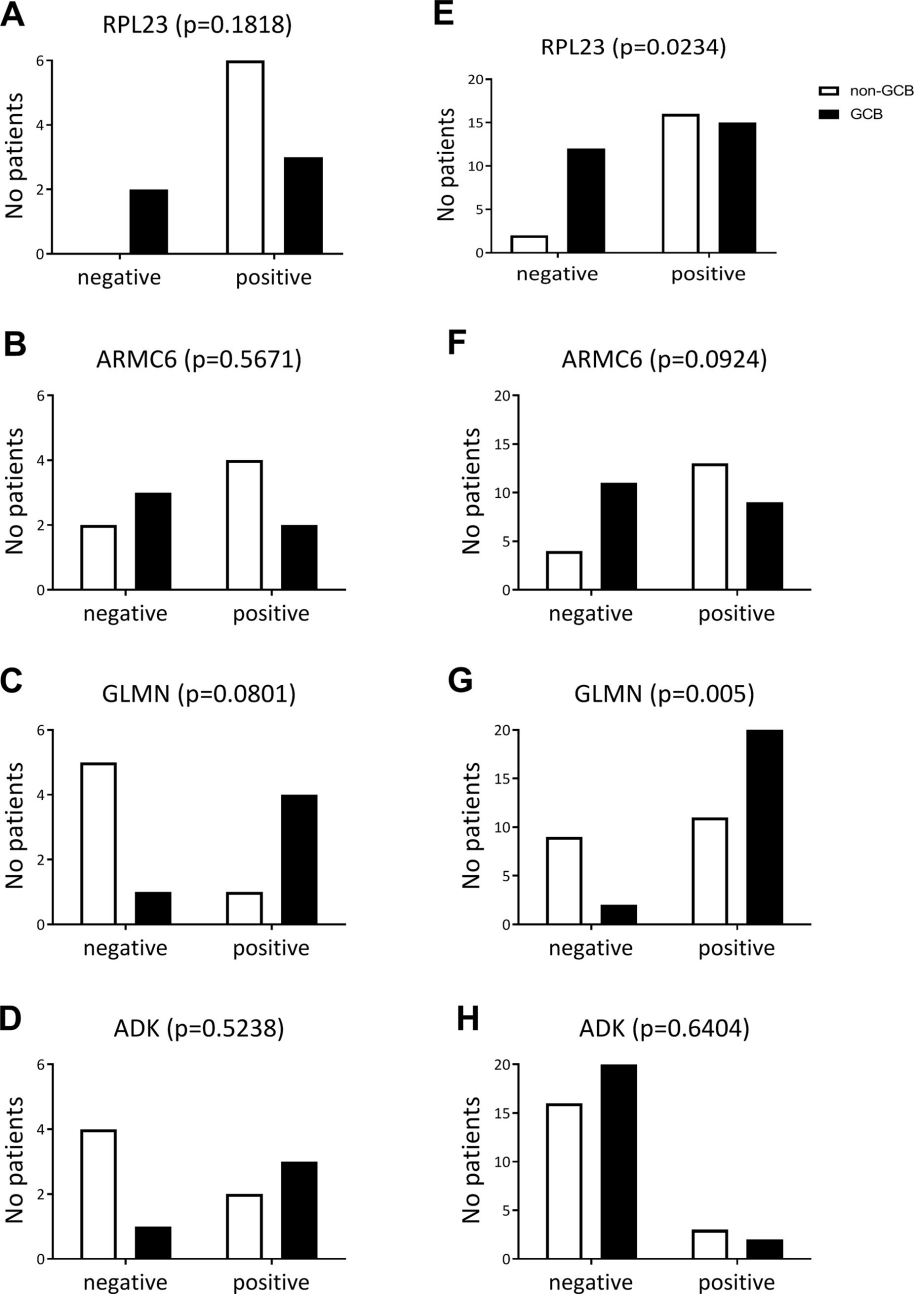
Results of the immunohistochemistry of the 4 selected proteins (RPL23, ARMC6, GLMN and ADK). **A.** Results of 10 cases with sufficient material for validation and **B.** results of the replication cohort of 47 independent DLBCL cases.

Validation of the proteomics results by immunohistochemistry was performed on 10 of the 12 super-SILAC cases. The paraffin blocks of two other cases did not contain sufficient tissue for the additional stainings. Representative images for each of the four antibodies are shown in (Fig 3) We observed trends that were similar to the proteomics results for all four proteins (Fig 4), but due to the low numbers of cases no definitive conclusions could be drawn.

### Immunohistochemical staining of the replication cohort

Replication was done on 47 independent DLBCL cases. Twenty cases were subtyped as non-GCB DLBCL and 27 cases as GCB DLBCL according to Hans classification. RPL23 showed significantly more positive cases (p = 0.0234) in non-GCB (89%) as compared to the GCB DLBCL cases (58%)(Fig 4). ARMC6 expression was observed at a higher frequency in non-GCB than in the GCB DLBCL group, albeit not significantly. Immunohistochemical validation of GLMN showed a significant difference (p = 0.005) with considerably more positive cases in GCB DLBCL (92%) than in non-GCB DLBCL (55%). The percentages of ADK positive cases was not different between GCB and non-GCB in our replication series. Thus, two of 4 proteins showed a significant difference consistent with the proteomics findings and discriminated between GCB and non-GCB DLBCL as defined by the Hans algorithm.

As an additional control, we also determined cell of origin using the Visco 3-thiered algorithm for a subset of the cases (for which we had enough material to stain FoxP1). We combined the validation and replication analysis, because of some drop outs. The staining patterns for the four tested proteins were very similar for the Hans and Visco algorithms (S3 Fig).

### Comparison with individual markers of the Hans criteria

To determine whether any of the markers identified in this study correlates with the individual markers used in the Hans algorithm, we investigated the total cohort of 58 cases (validation plus replication) (S1 Fig). Although not significant, there was a trend for RPL23 (p = 0.0578) and ARMC6 (p = 0.0730) expression in correlation with negative CD10 cases. No significant association were observed for GLMN and ADK.

### Correlation of RPL23 expression with p53 and MYC

As expression of RPL23 has been related to p53 and MYC in literature [19,20], we also stained the cases with sufficient material for these two proteins. For p53, 10 of 36 cases were homogeneous negative or positive and probably mutated and 26 cases showed variable staining and were considered wild type. Of these 18 and 8 were RPL23+, showing no correlation of p53 with RPL23 expression (S2 Fig).

For MYC 35 cases were stained and the percentage of MYC positive cells was compared to RPL23 expression (S2 Fig). RPL23 positive cases showed significantly higher MYC expression.

## Discussion

Using super-SILAC on membrane and cytoplasmic proteins of purified tumour cells isolated from primary viable cell suspensions we uncovered several proteins that were significantly differentially expressed between GCB and non-GCB DLBCL. We found no expression of CD10, similar to Deeb et al. who found only one peptide of CD10. [21] The lack of BCL6 in our analysis was likely due to the fact that the protein is almost exclusively localized in the nucleus, a compartment not thoroughly investigated in our study. Our analysis showed some differential expression of MUM1/IRF4, which is localized within the nucleus and cytoplasm, with slightly higher levels in non-GCB DLBCL. Two out of four proteins, i.e. RPL23 and GLMN, selected for validation and replication by immunohistochemistry showed a significantly differential expression pattern between GCB and non-GCB DLBCL consistent with the super-SILAC results. Comparison to the individual markers used for the Hans algorithm revealed a trend towards more positive RPL23 and ARMC6 positive cases in the CD10-negative cases, which confirms the importance of this marker in the Hans and Visco algorithms.

The total number of unique proteins identified in our study were similar to those in other proteomics studies. [9,10,22] Deeb et al. generated a super-SILAC mix with nine B-cell lymphoma lines and identified 6263 proteins in a super-SILAC analysis on 5 non-GCB DLBCL cell lines and 5 GCB DLBCL cell lines. They established a signature of 55 proteins that could differentiate between non-GCB DLBCL and GCB DLBCL subtypes. [9] Fifteen of the 55 proteins overlapped with the 4289 proteins found in our study and two proteins (CD81 and ARHGAP25) overlapped with our list of 132 proteins with at least 2 fold difference. In a second study Deeb et al. applied super-SILAC to differentiate between non-GCB DLBCL and GCB DLBCL using 20 FFPE DLBCL tissue samples. This revealed 5480 proteins with 343 differentially expressed proteins. The overlap with our total protein list (4289) was 199 proteins out of 343 proteins and the overlap with our list of 132 differentially expressed proteins was 6 out of 343 proteins respectively, CYB5R2, ANKRD13A, SUB1, SCRN1, ARHGAP25, NAP1L1. [10] Ruetschi et al. analysed frozen tissue sections of five relapsed and five long-term progression-free DLBCL patients using super-SILAC. The reference super-SILAC mix consisted of four DLBCL cell lines and one Burkitt lymphoma cell line. Of the 3588 proteins, 87 proteins were differentially expressed. Six (PSAP, PSMB7, PDCD4, ACTR2, CD44, MUM1) of the 87 proteins were found in our differentially expressed list. [11]

We followed a two-step approach to validate our findings with immunohistochemistry, validation in the same cases and replication in an independent cohort. We used The Hans and Visco algorithms to classify our cases. The Hans algorithm is the most commonly used approach to classify DLBCL cases in the routine diagnostic setting. [8] According to Visco et al. the Hans, Choi and Tally algorithms performed almost equally well and the 3- and 4-thiered Visco algorithms somewhat better compared to the gene expression based classifier. [23] We therefore decided to add the 3-thiered Visco algorithm to our study, for the validation and replication analyses of the four remaining proteins. Three of the 52 cases included switched from non-GCB to GCB or vice versa. Only minor differences were found between both algorithms indicating consistency of the differential expression pattern between both cell of origin subtypes.

The data on the validation cohort showed staining patterns that were consistent with the proteomics data, but the cohort was too small to perform meaningful statistical testing.

In the replication series significantly different expression that correlated with proteomics were found for 2 of 4 selected proteins, i.e. RPL23 and GLMN. RPL23 showed a more frequent staining in non-GCB than GCB DLBCL by both cell of origin classification approaches. Meng et al. studied the RAS–RPL23–MDM2–p53 pathway, and showed that increased levels of RPL23 induced by RAS were associated with increased p53 expression levels. [19] We investigated the relationship with p53 expression in the tumour cells of 36 cases that had been stained in our series but did not observe any differences (S2 Fig). RPL23 can also be induced upon activation of MYC [24]. MYC overexpression is observed in a considerable part of DLBCL, in particular in non-GCB DLBCL [25], and it is associated with a poor survival, in particular if combined with BCL2 protein overexpression. [25,26] Qi et al. observed MYC overexpression in combination with high levels of RPL23 in SKM-1, an acute myeloid leukaemia cell line. [24] We found a significantly higher expression of MYC in RPL23 positive cases consistent with the literature. In view of the positive association between MYC and RPL23 expression in our cases it might be suggested that RPL23 is regulated by MYC in DLBCL.

Expression of GLMN was more common in GCB DLBCL cases, both in the proteomics analysis and the replication study. GLMN is a FK506-binding protein (FKBP) associated protein, with two potential isoforms also known as FKBP associated protein 48 (FAP48) or FKBP associated protein 68 (FAP68). [26,27] In the DLBCL cell lines, we only observed the longer 68 kDa isoform. GLMN is part of the Skp1-Cullin-F-box-like complex and plays a role in the differentiation of smooth muscle cells. Loss of function mutations in GLMN in vascular smooth-muscle cells resulted in increased angiogenesis. [26,28] Expression of GLMN was uncommon in non-GCB DLBCL, both by proteomics and immunohistochemistry. This corroborates the increased angiogenesis as assessed by micro vessel density measurements in non-GCB DLBCL compared to GCB-DLBCL, which was linked to poor clinical outcome in non-GCB DLBCL. [29,30]

In conclusion, we performed super-SILAC on purified primary DLBCL tumour cells and showed a consistent differential expression pattern of two proteins between GCB and non-GCB type DLBCL. The two proteins identified by us could be incorporated in novel algorithms to discern GCB from non-GCB type DLBCL and perhaps to improve the prognostic significance of such algorithms (the present series being historical and not suitable for such analysis), but likely more important to better select DLBCL cases for specific mutation analyses and novel targeted therapies.

## Supporting information

S1 Fig Comparison of differentially expressed proteins for the three markers used in the Hans algorithm (all not significant). (TIF)

S2 Fig (A) RPL23 expression in relation to p53 expression in 36 DLBCL cases. (B) RPL23 expression in relation to immunohistochemical MYC expression (with mean) in the validation and replication cohort (57 DLBCL cases).
(TIF)

S3 Fig Comparison of differentially expressed proteins for the four markers used in the Hans algorithm and Visco. (TIF)

S1 Table Antibodies used for immunohistochemistry, antigen retrieval method and dilution. (DOCX)

S2 Table Flow cytometry analysis (FACS) to determine the purity of the DLBCL tumour samples used for the super-SILAC procedure. The percentage of remaining (reactive) polyclonal B cells was estimated using the percentage of lymphoid cells (within the lymphogate) with the other light chain isotype than the tumour cells and a kappa/ lambda ration of normal B cells of 3/2. In two cases (MM3 and MM7) high background levels prohibited accurate immunoglobulin light chain assessment (see text).
(DOCX)

S3 Table Super-SILAC ratios of five selected differentially expressed proteins per patient sample. For case 5 (GLMN) protein was not present in the analysis (-).
(DOCX)

S4 Table 132 proteins with at least a two-fold difference. (XLSX)
