# Supplementary figures and images for: A super-SILAC based proteomics analysis of diffuse large B-cell lymphoma-NOS patient samples to identify new proteins that discriminate GCB and non-GCB lymphomas
Source: PLoS One. 2019 Oct 11;14(10):e0223260. doi: 10.1371/journal.pone.0223260 (PMC6788715; doi:10.1371/journal.pone.0223260)

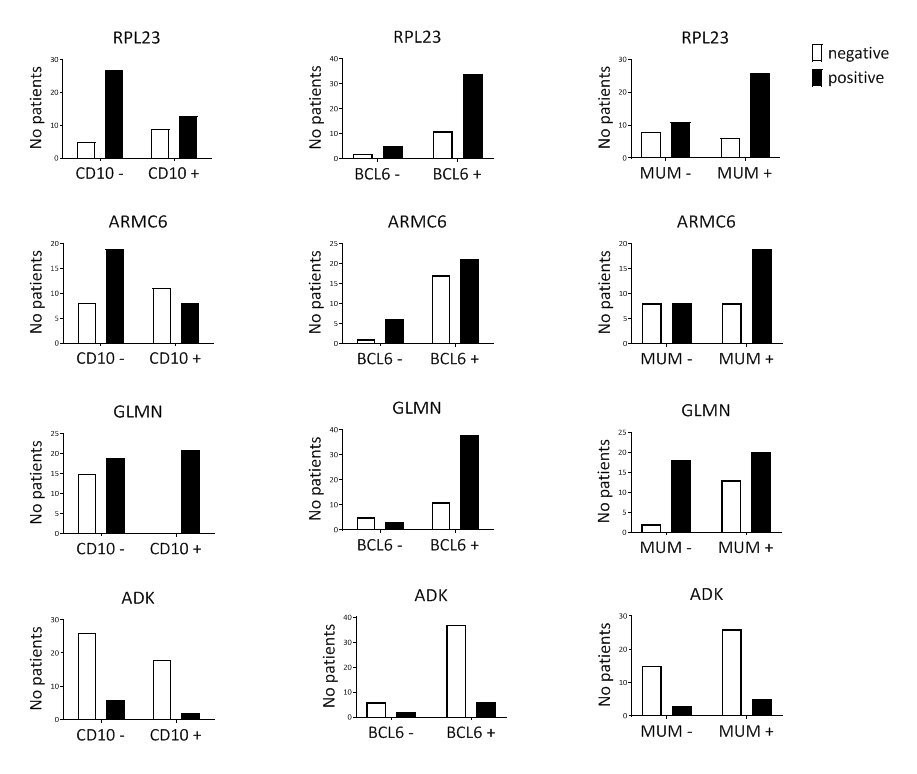

Supplement: S1 Fig — (TIF) [file pone.0223260.s001.tif]

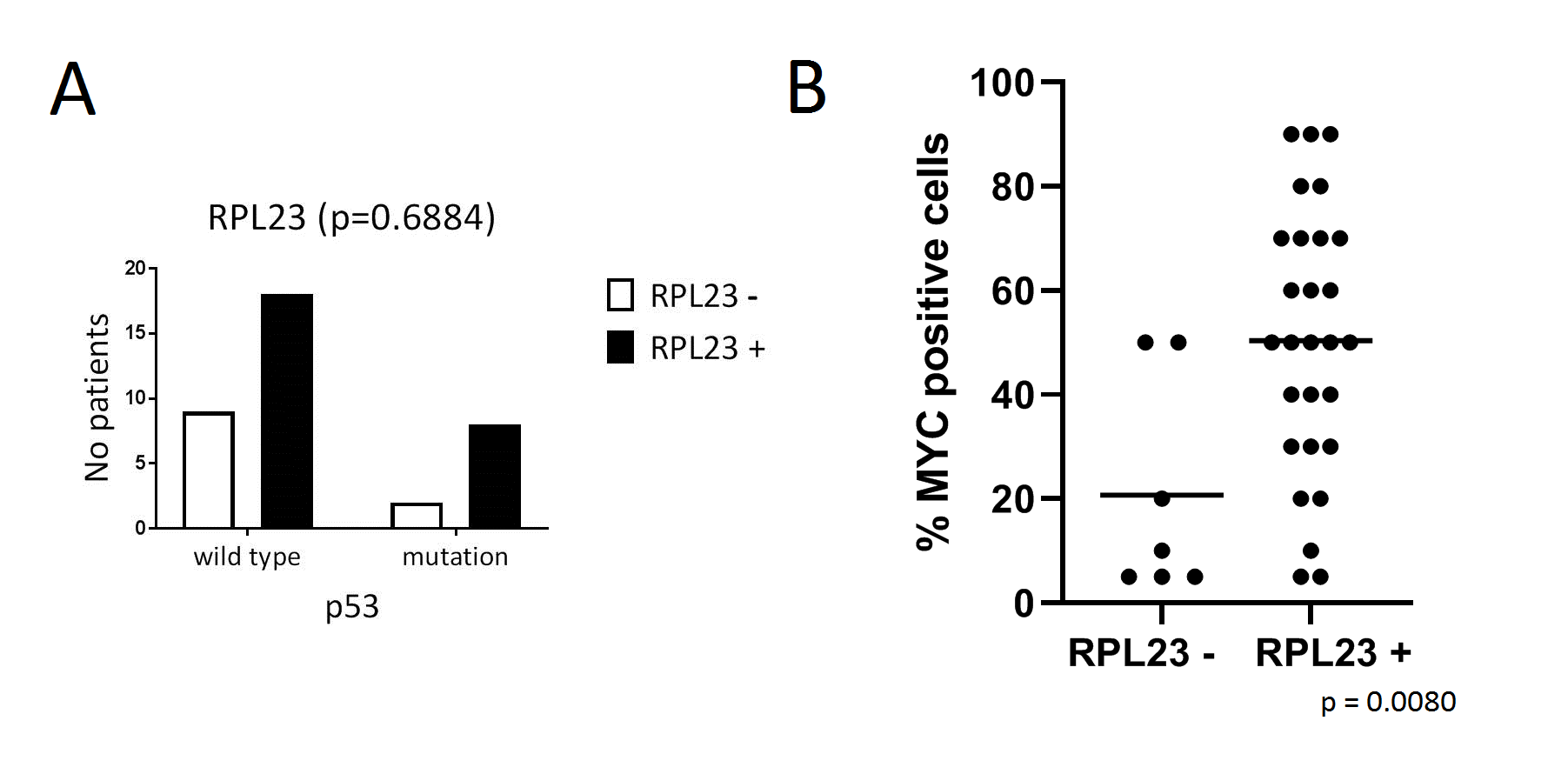

Supplement: S2 Fig — (A) RPL23 expression in relation to p53 expression in 36 DLBCL cases. (B) RPL23 expression in relation to immunohistochemical MYC expression (with mean) in the validation and replication cohort (57 DLBCL cases). (TIF) [file pone.0223260.s002.tif]

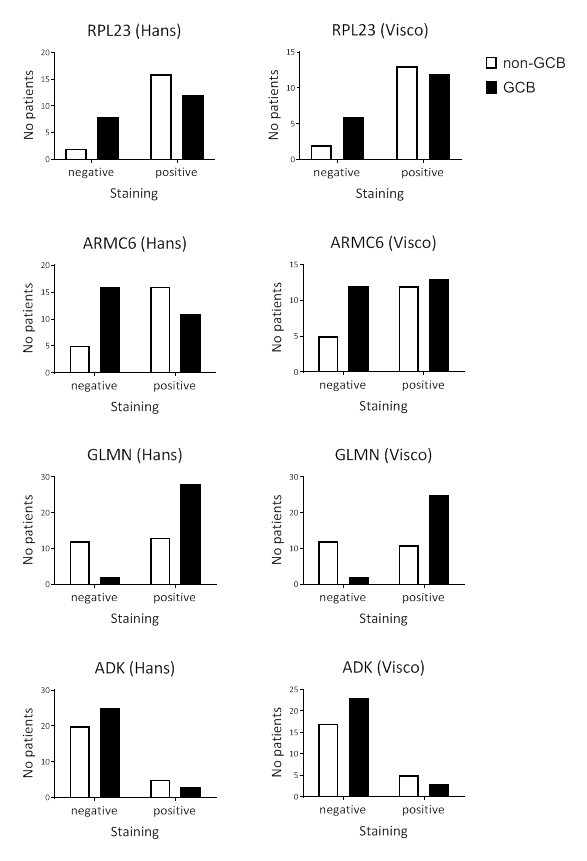

Supplement: S3 Fig — (TIF) [file pone.0223260.s003.tif]
