# Supplementary material for: A super-SILAC based proteomics analysis of diffuse large B-cell lymphoma-NOS patient samples to identify new proteins that discriminate GCB and non-GCB lymphomas
Source: PLoS One. 2019 Oct 11;14(10):e0223260. doi: 10.1371/journal.pone.0223260 (PMC6788715; doi:10.1371/journal.pone.0223260)
Supplement: S1 Table — (DOCX) [file pone.0223260.s004.docx]

**S1 Table.**

| **Antibody** | **Supplier** | **Antigen retrieval** | **Dilution** |
| --- | --- | --- | --- |
| GLMN | Abcam ab170776 | Tris 10 mM, pH10 | 1:40 |
| ADK | Atlas/SA HPA038391 | Citrate 10mM, pH9 | 1:20 |
| ARMC6 | Atlas/SA HPA041420 | Tris 10mM/EDTA 1 mM, pH9 | 1:100 |
| RPL23 | Atlas/SA HPA003373 | Citrate 10mM, pH9 | 1:50 |
