# Supplementary material for: A super-SILAC based proteomics analysis of diffuse large B-cell lymphoma-NOS patient samples to identify new proteins that discriminate GCB and non-GCB lymphomas
Source: PLoS One. 2019 Oct 11;14(10):e0223260. doi: 10.1371/journal.pone.0223260 (PMC6788715; doi:10.1371/journal.pone.0223260)
Supplement: S2 Table — The percentage of remaining (reactive) polyclonal B cells was estimated using the percentage of lymphoid cells (within the lymphogate) with the other light chain isotype than the tumour cells and a kappa/ lambda ration of normal B cells of 3/2. In two cases (MM3 and MM7) high background levels prohibited accurate immunoglobulin light chain assessment (see text). (DOCX) [file pone.0223260.s005.docx]

**S2 Table.**

| **Cases** | **CD20 (%)** | **sIg expression tumour cells** | **remaining polyclonal B cells (%)** |
| --- | --- | --- | --- |
| 1 | 98 | K | 23 |
| 2 | 99 | L | 10 |
| 3 | 96 | L? | ? |
| 4 | 98 | K | 45 |
| 5 | 98 | K | 0 |
| 6 | 85 | K | 5 |
| 7 | 98 | K | 20 |
| 8 | 99 | L | ? |
| 9 | 99 | K | 8 |
| 10 | 99 | K | 0 |
| 11 | 99 | K | 8 |
| 12 | 99 | L | 2 |
| 13 | 98 | K | 10 |
|  |  |  |  |

The percentage of remaining (reactive) polyclonal B cells was estimated using the percentage of lymphoid cells (within the lymphogate) with the other light chain isotype than the tumour cells and a kappa/ lambda ration of normal B cells of 3/2. In two cases (MM3 and MM7) high background levels prohibited accurate immunoglobulin light chain assessment (see text).
