# Supplementary material for: A super-SILAC based proteomics analysis of diffuse large B-cell lymphoma-NOS patient samples to identify new proteins that discriminate GCB and non-GCB lymphomas
Source: PLoS One. 2019 Oct 11;14(10):e0223260. doi: 10.1371/journal.pone.0223260 (PMC6788715; doi:10.1371/journal.pone.0223260)
Supplement: S3 Table — For case 5 (GLMN) protein was not present in the analysis (-). (DOCX) [file pone.0223260.s006.docx]

**S3 Table.**

| **Cases** | **Hans** | **GLMN** | **ADK** | **ARMC6** | **RPL23** |
| --- | --- | --- | --- | --- | --- |
| 1 | non-GCB | 0.11 | 0.11 | 14.95 | 5.91 |
| 2 | non-GCB | 0.13 | 0.09 | 6.96 | 5.38 |
| 3 | non-GCB | 1.29 | 0.76 | 1.32 | 26.58 |
| 5 | non-GCB | 5.78 | 0.28 | 3.55 | 6.53 |
| 6 | non-GCB | - | 0.58 | 7.29 | 5.70 |
| 7 | non-GCB | 0.11 | 0.84 | 17.87 | 0.83 |
| 8 | GCB | 12.19 | 9.06 | 0.24 | 0.21 |
| 9 | GCB | 31.95 | 0.86 | 0.39 | 0.14 |
| 10 | GCB | 10.88 | 7.33 | 1.40 | 0.07 |
| 11 | GCB | 4.74 | 7.89 | 0.17 | 0.32 |
| 12 | GCB | 8.79 | 2.36 | 0.40 | 0.11 |
| 13 | non-GCB | 14.60 | 1.18 | 0.27 | 37.34 |
|  |  |  |  |  |  |

For case 5 (GLMN) protein was not present in the analysis (-).
